# The role of clinical pilates exercises in children wıth juvenile idiopathic arthritis: a pilot study

**DOI:** 10.1186/1546-0096-9-S1-P117

**Published:** 2011-09-14

**Authors:** Edibe Unal, Pinar Dizmek, Yelda Bilginer, Asli Celebi Tayfur, Nesrin Besbas, Seza Ozen

**Affiliations:** 1 Hacettepe University,Faculty of Health Science, Ankara, Turkey; 2 Hacettepe University, Faculty of Medicine, Ankara, Turkey

## Background

Juvenile İdiopathic Arthritis(JIA) affects the quality of life to a significant degree causing the child to become less social.The family puts on an overprotective attitude. The importance of exercise is remarkable in these children. Clinical Pilates Exercises don’t generate fatigue and help inhibition of pain with respiratory control. This exercise is entertaining, and can be applied as a group. Even though pilates is used on adult rheumatic patients, there is no study in JIA.

## Aim

To investigate the effects of Clinical Pilates Exercises on functional status,pain level, and general health perception in children with JIA.

## Methods

The study included 23 children(14F;9M) with JIA. After obtaining demographics, Time-up&Go test (TUG), Time to go&back(10m)(GGT), the number of Sit-Stand up in 30 seconds, FACES Pain Scale, the child, family and physiotherapist global assesment Visual Analogue Scale(VAS), Childhood Health Assessment Questionnaire (CHAQ) was performed in all. Sedimentation rate (ESR) and C-reactive protein(CRP) were recorded. The same assessments were repeated after treatment. Therapy sessions were done thrice a week. The session included warm-ups, the main workout and cool down. The exercises consisted of clinical pilates exercises,dancing and body image affirmation exercises.

## Results

The mean age was 11,46±2,84years. The mean duration of disease was 4,75±2,2years. 13 oligoarticuler and 10 polyarticular type JIA included. After the treatment, there were significant improvements in the Sit-Stand up test, FACES, ESR, CRP, the child and physiotherapist global assesment VAS scores (P<0,05). TUG, GGT, CHAQ, the family global assesment VAS scores didn’t change significantly(P>0,05).

## Conclusion

Clinical pilates exercises have shown positive effects on decreasing the pain, improving function and general health preception. Clinical Pilates Exercises can be considered as reliable exercise model in children with JIA.

